# From data to action: leveraging Global Burden of Disease Studies for rheumatic and musculoskeletal diseases

**DOI:** 10.3389/fimmu.2025.1754256

**Published:** 2026-02-10

**Authors:** Shudan Chen, Yueyang Liu, Yu Xin, Yifan Qiu, Xuqiang Geng, Jiafeng Zhang

**Affiliations:** 1 Department of Endocrinology, No. 905 Hospital of People's Liberation Army (PLA) Navy, Shanghai Changzheng Hospital, Shanghai, China; 2 School of Basic Medical Sciences, Naval Medical University, Shanghai, China; 3 Department of Plastic Surgery, The People's Hospital of Yingkou, Yingkou, Liaoning, China; 4 Department of Gastroenterology, Changhai Hospital, Naval Medical University, Shanghai, China; 5 Department of Rheumatology and Immunology, Shanghai Changzheng Hospital, Naval Medical University, Shanghai, China

**Keywords:** disability-adjusted life years, Global Burden of Disease, health equity, rheumatic and musculoskeletal diseases, rheumatoid arthritis

## 1 Introduction

Rheumatic and musculoskeletal diseases (RMDs), encompassing conditions from rheumatoid arthritis (RA) and systemic lupus erythematosus (SLE) to spondyloarthritis (SpA) and systemic sclerosis, pose a major global challenge (1). Although mortality rates for many RMDs remain relatively low, their chronicity, disability burden, and societal impact are profound (2).

From the perspective of practicing rheumatologists, the burden of RMDs is not merely reflected in epidemiological curves, but in delayed diagnoses, irreversible joint or organ damage, prolonged pain, and loss of functional independence encountered daily in clinical practice. Many patients present years after symptom onset, often after navigating fragmented referral pathways—particularly in low- and middle-income countries (LMICs). These lived clinical realities underscore the need for population-level data frameworks that can quantify burden while also informing earlier, more equitable intervention strategies.

The Global Burden of Disease (GBD) Study, developed and coordinated by the Institute for Health Metrics and Evaluation (IHME), has provided our field with an unprecedented macro-lens: a standardized, global, temporally and demographically resolved map of disease burden (3). This editorial argues that integrating GBD−derived data into the RMDs ecosystem is no longer optional — it is essential for research prioritization, clinical translation, policy advocacy and global health equity.

## 2 Why GBD matters to RMDs

RMDs share several key epidemiologic features that make population-level burden data particularly valuable. These conditions are characterized by chronicity, prolonged morbidity rather than acute mortality, marked geographic and socio-demographic heterogeneity, and requirements for long-term management (4). Traditional registry-based studies and clinical cohorts, while invaluable for mechanistic insights, tend to be confined to specialist centers or high-income settings, often lacking cross-country comparability or long-term population-scale trend data.

In contrast, the GBD framework delivers standardized metrics — incidence, prevalence, mortality, years lived with disability (YLDs), years of life lost (YLLs), and disability-adjusted life years (DALYs) — across more than 200 countries and territories, spanning multiple years and stratified by age, sex, and sociodemographic index (SDI) (5, 6). For the RMDs field, this comprehensive approach enables identification of which conditions impose the greatest burden globally, assessment of how that burden is evolving across regions and socioeconomic strata, and determination of where health disparities are most apparent and where resources should be directed.

## 3 Insights from the GBD data

### 3.1 Quantifying the Global Burden: RA as exemplar

RA is the most mature RMD in terms of burden‐data. The GBD 2021 report revealed that global age-standardized prevalence rates (ASPR) of RA increased from 1990 to 2020, while the age-standardized DALY rate (ASDR) declined slightly (6). Another study showed that for those aged 60 and older, age-standardized incidence rate (ASIR) rose from 24.9 to 30.3 per 100,000 between 1990 and 2021, and ASPR increased from 635.5 to 726.9 (7). These data highlight two concurrent trends: improved detection and demographic changes (leading to higher prevalence and incidence), as well as advancements in therapy and management (resulting in reduced mortality) — yet persistent disability remains a significant issue. **Table 1** presents comparative burden metrics for major RMDs.

**Table 1:** Global Burden comparison of major rheumatic and immunological diseases (GBD 1990-2021).

Disease	Global prevalence (millions)	Age-standardized prevalence rate (/100,000)	DALYs (millions)	Age-standardized DALY rate (/100,000)	Time trends (1990–2020)	Source
Rheumatoid Arthritis	17.6 (15.8-20.3)	208.8 (186.8-241.1)	3.06 (2.32-3.86)	36.4 (27.6-45.9)	ASPR↑ 14% ASDR↓ 23.8%	(9)
Osteoarthritis	595 (535–656)	6967.3 (6180.7-7686.1)	21.7 (10.2-47.6)	255 (119.7-557.2)	ASPR↑ 14.1%, DALY↑ 134%	(15)
Gout	55.8 (44.4-69.8)	659.3 (525.4-822.3)	1.73 (1.22-2.39)	20.5 (14.4-28.2)	ASPR↑22.5% DALY↑ 22%	(16)
Other MSK Diseases*	494 (431–564)	5910 (5180–6750)	44.91 (31.61-62.42)	539 (380–744)	ASPR↑ 25.2% ASDR↑ 11.1%	(10)

ASPR, age-standardized prevalence rate; DALY, disability-adjusted life year; MSK, musculoskeletal.

*Other MSK diseases include systemic lupus erythematosus, ankylosing spondylitis, psoriatic arthritis, systemic sclerosis, Sjögren’s syndrome, and other autoimmune conditions.

GBD classification groups several distinct autoimmune diseases under “Other MSK,” which may underestimate individual disease burdens.

The clinical significance of these epidemiological trends cannot be overstated. Rheumatologists recognize a critical “window of opportunity” — typically the first 3–6 months after symptom onset — during which aggressive treatment can fundamentally alter disease trajectory and prevent irreversible joint damage (8). The rising incidence rates revealed by GBD data, particularly in aging populations, translate directly into increased demand for early rheumatology access. Yet in many healthcare systems, the average time from symptom onset to rheumatology consultation exceeds 6 months, effectively closing this therapeutic window for countless patients.

### 3.2 Revealing macro-inequalities and shifting burdens

GBD data systematically reveal regional disparities in the burden of RA. Age-standardized RA prevalence is highest in Andean Latin America (432.76 per 100,000) and lowest in Oceania (50.75 per 100,000), reflecting complex interactions of genetic, environmental, and healthcare access factors (4). Concerningly, mortality trends also show significant inequalities: RA mortality has declined by 43.8% in high-income countries, while it has increased by 530.6% in Central Asia and by 6.4% in Eastern Europe (9). These disparities reflect differences in treatment accessibility, particularly early access to disease-modifying antirheumatic drugs (DMARDs) and biologics.

These regional inequalities have profound clinical consequences beyond what aggregate statistics capture. In resource-limited settings, rheumatologists frequently encounter patients presenting with advanced joint destruction, fixed deformities, or organ damage that would be preventable with timely diagnosis and treatment. The absence of pediatric rheumatology services in many LMICs means that children with juvenile idiopathic arthritis may face lifelong disability from growth abnormalities and joint contractures. These clinical realities remind us that behind every DALY statistic lies an individual whose quality of life hangs in the balance.

The Socio-Demographic Index (SDI) analysis reveals that the burden of RMDs is shifting toward lower-resource settings. Middle-low SDI regions show the fastest annual percentage change (AAPC), yet these regions often lack the capacity for adequate disease management. Qualitative research in 29 African countries has identified key barriers to effective management, including inconsistent methotrexate supply, limited healthcare providers, financial constraints, and inadequate safety monitoring systems. Biologics are particularly difficult to introduce in low-income countries due to high costs (9).

### 3.3 Identifying research priorities and knowledge gaps

GBD data enable evidence-based research prioritization by identifying which diseases are increasing most rapidly, in which regions, and among which populations. For instance, these data can direct resources toward early detection of spondyloarthritis in low- and middle-income regions or address the growing burden of psoriatic arthritis in aging populations. The observed regional variations in incidence trends also prompt mechanistic investigations into the roles of environmental exposures, lifestyle factors, microbiome composition, and genetic susceptibility. Furthermore, burden metrics can help define clinical trial priorities and advocate for resource allocation aligned with high-burden conditions.

GBD data also expose critical knowledge gaps. Current RA estimates derive from only 45 of 204 countries, covering 16 of 21 GBD regions (9). For other musculoskeletal disorders (MSK), data are derived from only 68 sources across 23 countries (10). This metric — years lived with disability (YLD) — is particularly important for RMDs, as these conditions cause substantial long-term functional impairment despite relatively low mortality. Geographic data scarcity is especially evident in low- and middle-income countries (LMICs), where comprehensive musculoskeletal policy documents are often absent (11). These gaps identify priority regions for epidemiological research, particularly sub-Saharan Africa, South Asia, and Southeast Asia.

### 3.4 Policy translation and health system planning

**Figure 1:**
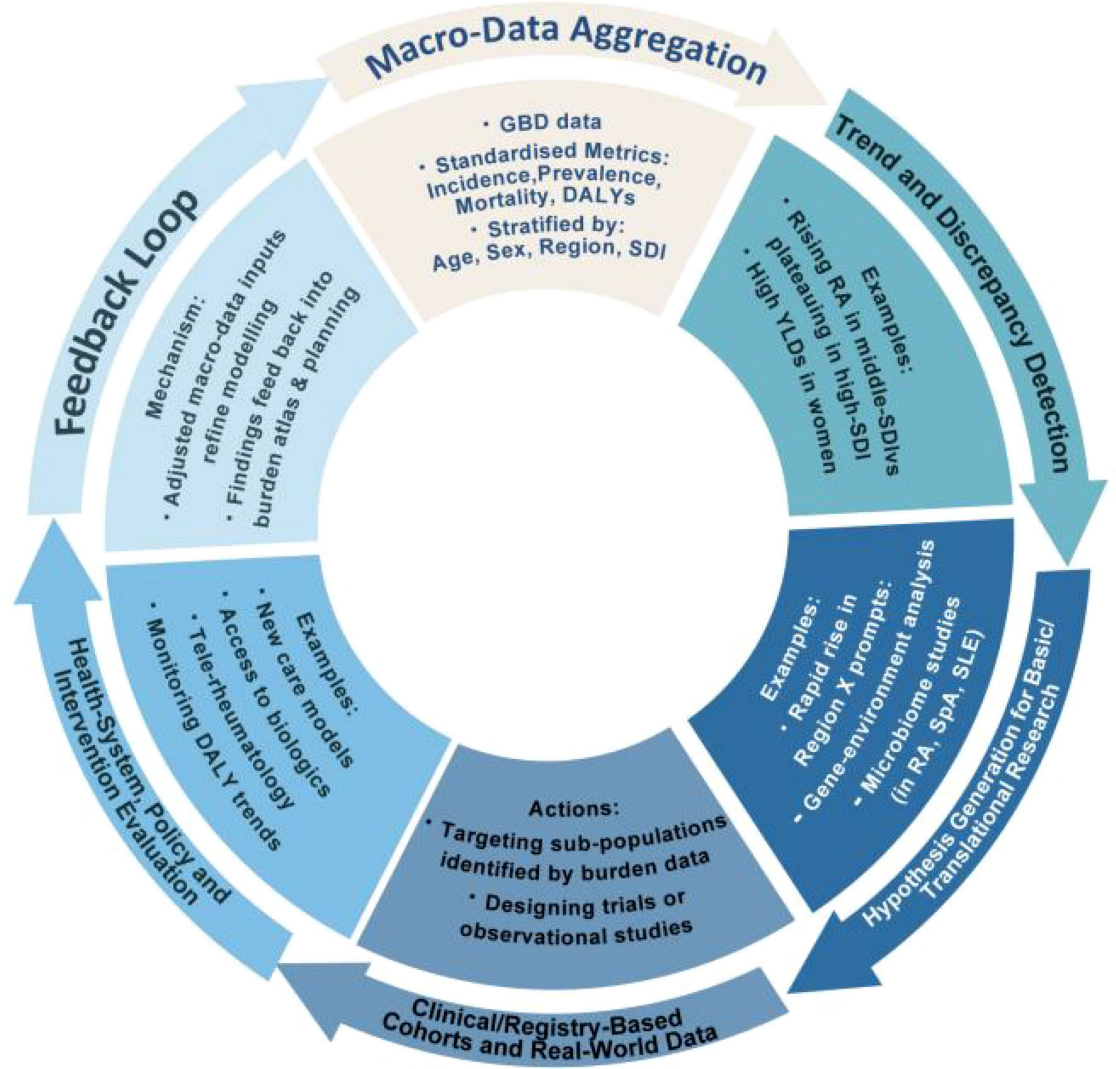
Translational cycle: from burden data to intervention impact. GBD, Global Burden of Disease; DALYs, Disability-Adjusted Life Years; SDI, Sociodemographic Index; YLDs, Years lived with Disability; MSK, Musculoskeletal; RA, Rheumatoid Arthritis; SpA, Spondyloarthritis; SLE, Systemic Lupus Erythematosus. This schematic illustrates how GBD platforms facilitate translation from macro-level epidemiological data through mechanistic research to clinical interventions, with burden metrics providing feedback for continuous improvement in research prioritization and policy development.

For policymakers and global health stakeholders, burden metrics provide the language of impact: DALYs lost, YLDs accumulated, and economic consequences of disability. Demonstrating that RA or SpA consumes substantial population health resources strengthens advocacy for early-diagnosis programs, DMARD/biologic access, rehabilitation services, and workforce development. The GBD platform’s regional estimates facilitate national policy planning and international benchmarking. **Figure 1** illustrates how burden data platforms support the translational cycle from epidemiological insight to intervention impact.

## 4 Opportunities and challenges ahead

While the GBD platform has already contributed significantly, the RMDs community now faces both strategic opportunities and responsibilities. Several areas warrant urgent attention:

### 4.1 Expanding disease scope

Most GBD outputs emphasize RA, osteoarthritis (OA) or broad musculoskeletal categories. Many classic systemic RMDs — such as SLE, systemic sclerosis, primary Sjögren’s syndrome, vasculitides, and juvenile idiopathic arthritis — are poorly captured or absent in routine burden modeling. Expanding the disease scope and improving data input is imperative.

### 4.2 Improving data quality and coverage

Modeled burden estimates are only as good as the underlying data. Many LMICs lack rheumatology or dermatology registries, comprehensive national surveillance systems, or reliable diagnostic tools (12, 13). This leads to uncertainty and potentially underestimates the burden in underserved regions. Global capacity-building in rheumatology epidemiology is essential.

### 4.3 Bridging macro and micro perspectives

The GBD provides descriptive epidemiology of burden but stops short of proving causality or intervention effect. The next phase for the RMDs field should focus on linking burden metrics with mechanistic research (genomics, immunology, microbiome, environment factors) and intervention trials or health-system studies that track changes in burden. Real-world data from rheumatology registries could contribute to future GBD iterations.

### 4.4 Monitoring intervention impact

In fields such as cardiology and oncology, declines in DALYs have corresponded with wide adoption of therapies and prevention programs (14). In RMDs, translating therapy uptake — such as early DMARD/biologic use in RA or treat-to-target strategies in spondyloarthritis — into population-level burden reduction remains underexplored. Designing studies where national care models are aligned with tracking burden metrics offers a powerful evaluation framework.

### 4.5 Leveraging digital health and AI

Emerging opportunities include integrating artificial intelligence with GBD data for predictive modeling, utilizing digital health platforms for real-time surveillance, and expanding tele-rheumatology to underserved regions.

### 4.6 Advancing health equity

GBD data consistently highlight gender, age, and socioeconomic disparities. For RMDs, this translates into actionable imperatives: improving access to rheumatology services in LMICs, addressing diagnostic delays in women and older adults, expanding tele-rheumatology, and leveraging burden data for advocacy. Burden metrics provide the evidence base for global health negotiations.

### 4.7 Strengthening multidisciplinary care models

Modern rheumatology care extends beyond pharmacotherapy to encompass physical therapy, occupational therapy, psychological support, and patient education. The disability burden captured by YLDs reflects not only disease activity but also the adequacy of comprehensive care. GBD data can inform health system planning by quantifying the rehabilitation and allied health professional needs in different regions. Rheumatologists increasingly recognize that optimal outcomes require integrated care teams — a perspective that population-level burden data should inform and support.

## 5 Conclusion

For the global rheumatology and immunology community, GBD data have moved from being “nice to have” to fundamental infrastructure. These data offer a macro-view of where disease burden lies, how it is shifting, and where inequities persist. In a field characterized by chronicity, heterogeneity, disability and global variation, this macro−lens is indispensable.

The current moment presents both opportunity and responsibility. We propose three concrete actions: first, establishing a global RMDs registry network to improve data input for future GBD iterations; second, advocating that the next GBD cycle incorporate more systemic autoimmune diseases as distinct categories; and third, encouraging national rheumatology societies to integrate burden metrics into policy advocacy toolkits. The goal is not merely to enumerate burden but to reduce it. The rheumatology community now has a seat at the table of global health metrics—it is incumbent upon us to translate these data into action for patients today and populations tomorrow.

## Glossary

AAPC: Annual Percentage Change
AI: Artificial Intelligence
ASDR: Age-Standardized DALY Rate
ASIR: Age-Standardized Incidence Rate
ASPR: Age-Standardized Prevalence Rate
DALYs: Disability-Adjusted Life Years
GBD: Global Burden of Disease
IHME: Institute for Health Metrics and Evaluation
LMICs: Low- and Middle-Income Countries
MSK: Musculoskeletal
OA: Osteoarthritis
RA: Rheumatoid Arthritis
RMDs: Rheumatic and Musculoskeletal Diseases
SDI: Sociodemographic Index
SLE: Systemic Lupus Erythematosus
SpA: Spondyloarthritis
YLDs: Years Lived with Disability
YLLs: Years of Life Lost
